# Renal ischemic adverse drug events related to tranexamic acid in women of child-bearing age: an analysis of pharmacovigilance data

**DOI:** 10.1007/s00228-020-03064-y

**Published:** 2020-12-19

**Authors:** Dominik Stämpfli, Stefan Weiler, Carolyn F. Weiniger, Andrea M. Burden, Michael Heesen

**Affiliations:** 1 Pharmacoepidemiology, Institute of Pharmaceutical Sciences, Department of Chemistry and Applied Biosciences, ETH Zurich, Vladimir-Prelog-Weg 4, CH-8093 Zürich, Switzerland; 2 National Poisons Information Centre, Tox Info Suisse, Associated Institute of the University of Zurich, Zürich, Switzerland; 3 Division of Anesthesia, Critical Care and Pain, Tel Aviv Sourasky Medical Center, Tel Aviv, Israel; 4 Department of Anesthesia, Kantonsspital Baden, Baden, Switzerland

**Keywords:** Tranexamic acid, Renal cortical necrosis, Obstetrics, Post-partum hemorrhage

## Abstract

**Purpose:**

In response to a large trial, the World Health Organization broadened their recommendation on tranexamic acid to be used for post-partum hemorrhage. A 2013 French periodic safety update report warned of an abnormally high rate of renal cortical necrosis associated with tranexamic acid and other drugs for severe post-partum hemorrhage. We aimed to identify the reporting incidence of adverse thrombo-embolic events among women in child-bearing age who received tranexamic acid, with a focus on renal vascular and ischemic conditions.

**Methods:**

We analyzed individual case safety reports (ICSRs) on renal vascular and ischemic conditions, pulmonary thrombotic and embolic conditions, and peripheral embolism and thrombosis from the database of the World Health Organization – Uppsala Monitoring Centre (WHO-UMC). ICSRs were restricted to reports including tranexamic acid as a suspected drug, sex reported as female, and reported age between 18 and 44 years. Reporting odds ratios (RORs) and 95% confidence intervals (95% CIs) were calculated by comparing ICSRs on tranexamic acid to all other drugs in VigiBase.

**Results:**

Within 2245 included ICSRs on tranexamic acid, we identified 29 reports of adverse renal vascular and ischemic conditions, 42 reports of pulmonary thrombotic and embolic conditions, and 41 reports of peripheral embolism and thrombosis. RORs were statistically significant by 32.6-fold (32.62, 95% CI: 22.50–47.29), 2.5-fold (2.52, 95% CI: 1.85–3.42), and 2.7-fold (2.67, 95% CI: 1.96–3.64), respectively, when compared to any other drug within VigiBase.

**Conclusion:**

Tranexamic acid might bear an increased risk for renal ischemic adverse drug events in women of child-bearing age.

## Introduction

Tranexamic acid, an inhibitor of fibrinolysis, was recently recommended by the World Health Organization (WHO) for post-partum hemorrhage [1]. This was an update to the 2012 WHO recommendations where use was restricted to situations of failure of uterotonics or to a birth trauma as a cause of bleeding [2]. The updated recommendations broadening the indication followed the WOMAN (World Maternal Antifibrinolytic) trial [3], which was a large multicenter trial in high-resource as well as in low-resource settings. The trial revealed a significant reduction in hemorrhage-related mortality with early use of tranexamic acid (1 to 3 h) and no increase of adverse drug events.

A recent review of post-partum hemorrhage guidelines from developed countries and organizations around the globe found that the Royal College of Obstetricians and Gynaecologists (RCOG) was the only official obstetric body recommending, in 2017, against the use of tranexamic acid [4]. However, a 2019 RCOG document on maternal collapse now recommends the use of tranexamic acid, which is in line with clinical practice in the UK that shifted to using tranexamic acid [5]. The meta-analysis by Gayet-Ageron and colleagues [3, 6] and the WOMAN trial [3] support the efficacy of tranexamic acid for treating post-partum hemorrhage, while a randomized controlled trial by Sentilhes and colleagues [7] did not find a benefit of prophylactic tranexamic acid after vaginal delivery.

Although the efficacy of tranexamic acid is widely appreciated, less is known about the safety. A meta-analysis on randomized controlled trials (RCTs) concluded that tranexamic acid was not associated with an increased risk of adverse events compared to placebo or no treatment [8]. However, the number of obstetric trials in this analysis was low (6%) and included only 3251 patients. A French periodic safety update report (PSUR) in 2013 warned of an abnormally high rate of unexplained kidney failure after administration of tranexamic acid together with other drugs for severe post-partum hemorrhage [9]. Three years later, another French study reported 18 cases of renal cortical necrosis in women with post-partum hemorrhage who had received tranexamic acid [10]. At 6 months after delivery, none had recovered normal renal function and eight of the 18 patients (44%) were dialysis dependent. Length of exposure to tranexamic acid was the only risk factor which was associated with poorer kidney outcomes—in contrast to other studied variables such as blood loss volume, disseminated intravascular coagulation, hemodynamic instability, and exposition with other nephrotoxins.

In light of the limited evidence, we sought to assess renal ischemic adverse events associated with the use of tranexamic acid for post-partum hemorrhage reported to the World Health Organization – Uppsala Monitoring Centre (WHO-UMC) database on spontaneous reports of adverse drug events. We aimed to identify the reporting incidence of adverse thrombo-embolic events among women in child-bearing age who received tranexamic acid with a focus on renal vascular and ischemic conditions.

## Methods

### Database and study population

Data on individual case safety reports (ICSRs) were retrieved from WHO-UMC VigiBase. VigiBase contains spontaneous reports on adverse drug events sent by member countries of the WHO Programme for International Drug Monitoring. The original reports, submitted by clinicians, manufacturers, and patients to national pharmacovigilance centers, are amended with the standard terminology MedDRA® for the reported adverse events [11]. The included drugs are given their WHO Anatomical Therapeutical Chemical (ATC) classification [12]. Aggregated data on ICSRs available through the interface VigiLyze contain variables on identifiers, report properties, reporter qualification, patient age and sex, reported adverse events, reported outcomes, and reported drug regimen (substance, dose, regimen, route, starting, and stopping date). Causality and clinical assessments are handled differently by the various national pharmacovigilance centers.

All ICSRs with tranexamic acid (ATC: B02AA02) reported as a suspected, concomitant, or interacting drug were identified among those with sex reported as female. ICSRs were then restricted to reports describing tranexamic acid as suspected drug with patients’ age reported between 18 and 44 years. ICSRs with missing sex or age were excluded.

Adverse events of interest were defined as the preferred terms included within the MedDRA® high-level terms “renal vascular and ischemic conditions,” “pulmonary thrombotic and embolic conditions,” and “peripheral embolism and thrombosis.” Number of events was defined as the number of unique ICSRs, as identified by the WHO-UMC report ID.

### Statistical analysis

Demographic characteristics of the ICSRs were summarized using means and standard deviations and counts and proportions where appropriate, stratified by the three terms of interest. For renal vascular and ischemic conditions, we additionally investigated dosing of tranexamic acid and outcomes of reported events.

As a measure of disproportionality within the database, reporting odds ratios (RORs) with corresponding 95% confidence intervals (95% CIs) were calculated [13]. RORs compare the number of reports mentioning the drug of interest and the adverse drug event of interest to the reports for the outcome of interest among all other drugs in the database [14]. For this study, the ratio of reports on “renal vascular and ischemic conditions,” “pulmonary thrombotic and embolic conditions,” and “peripheral embolism and thrombosis” with tranexamic acid to with any other drug built the nominator, while the ratio of reports on any other event with tranexamic acid to with any other drug built the denominator. All analyses were performed in RStudio, version 1.2.5019 or later [15], running R, version 3.6.0 or later [16], and the packages data.table [17], dplyr [18], tidyr [19], and lubridate [20].

## Results

### Pharmacovigilance database

Data on tranexamic acid were extracted on October 21, 2019. ICSRs on tranexamic acid and female sex amounted to 5328 ICSRs from first database entry (June 30, 1972) until date retrieved (latest: October 19, 2019). The dataset was reduced to 5261 ICSRs by restriction to reports describing tranexamic acid as suspected drug and, finally, to 2245 ICSRs by restriction to reported ages 18 to 44 years. The original dataset of 5328 ICSRs contained 328 missing values on age (6.2%), which were excluded.

**Fig. 1:**
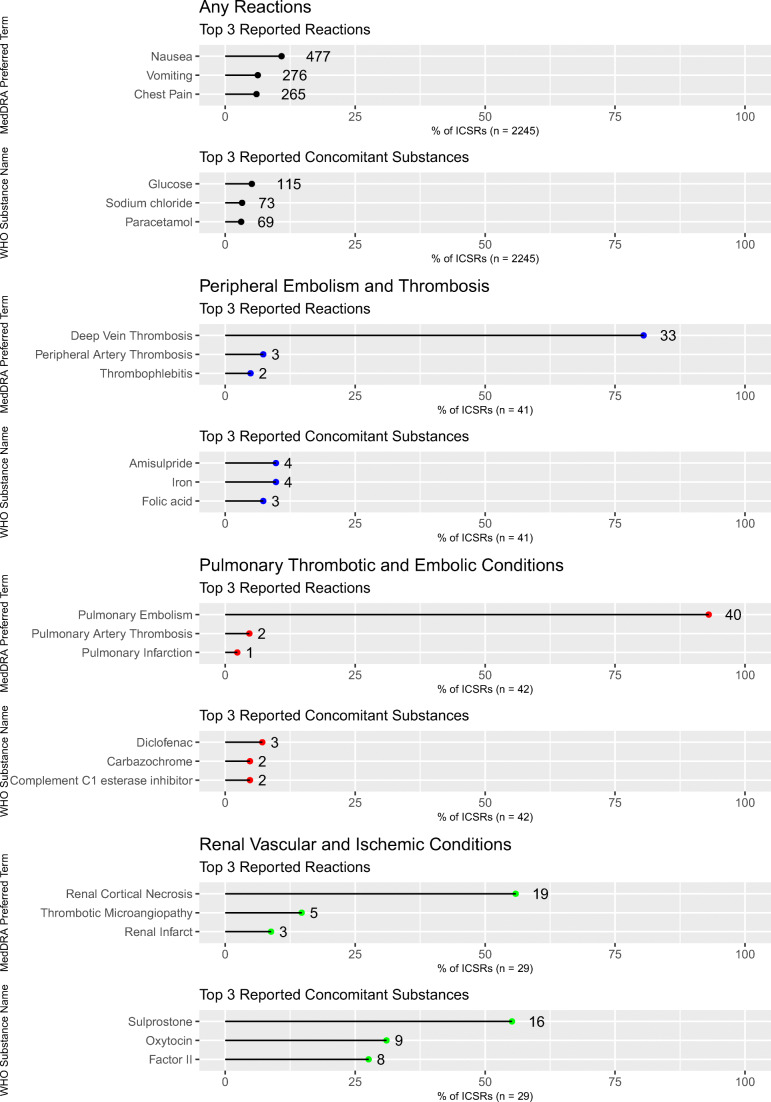
Top three reported concomitant medication and reported reactions within individual case safety reports (ICSRs) on tranexamic acid in VigiBase, stratified by all and the events of interest. ICSRs were filtered for tranexamic acid reported as suspected drug, female sex, and reported age 18 to 44 years. Numbers may not add up to 100%: An ICSR may report more than one concomitant medication

Table 1 summarizes report characteristics stratified by the three terms of interest. We identified 29 reports of adverse renal vascular and ischemic conditions (1.3%), 42 reports of pulmonary thrombotic and embolic conditions (1.9%), and 41 reports of peripheral embolism and thrombosis (1.8%). Overall, the reported mean age was 33.4 years (standard deviation: 7.3). Mean age was highest for peripheral embolism and thrombosis (37.3 ± 5.6 years) and lowest for renal vascular and ischemic conditions (32.3 ± 5.0 years). Year of first database entry was different for all three terms of interest and ranged from 1980 for peripheral embolism and thrombosis to 2014 for renal vascular and ischemic conditions. Reported mortality was highest for pulmonary thrombotic and embolic conditions. Figure 1 shows the top three of reported concomitant medication and reported reactions, stratified by all and the three events of interest. Reported medication regimen was different for renal vascular and ischemic conditions, pulmonary thrombotic and embolic events, and peripheral embolism and thrombosis, respectively.

**Table 1:** Characteristics of individual case safety reports (ICSRs) on tranexamic acid in VigiBase, stratified by the events of interest. ICSRs were filtered for tranexamic acid reported as suspected drug, female sex, and reported age 18 to 44 years. Numbers are not mutually exclusive

Characteristics	Any	Peripheral embolism and thrombosis	Pulmonary thrombotic and embolic conditions	Renal vascular and ischemic conditions
Number of reports	2245	41	42	29
First database entry	March 31, 1973	December 31, 1980	March 31, 1990	October 17, 2014
Mean age, (years ± SD)	33.4 ± 7.3	37.3 ± 5.6	34.2 ± 6.8	32.2 ± 5.0
Reported outcome				
Death	19 (0.8%)	< 5	5 (11.6%)	< 5
Not recovered	135 (5.8%)	8 (19.5%)	< 5	< 5
Recovered with sequelae	32 (1.4%)	< 5	< 5	5 (14.7%)
Recovering^†^	646 (27.9)	< 5	5 (11.6%)	< 5
Recovered	1137 (49.1%)	18 (43.9%)	16 (37.2%)	10 (29.4%)
Death unrelated to reaction	< 5	< 5	< 5	< 5
Unknown	221 (9.6%)	< 5	22 (4.7%)	12 (35.3%)
Not reported	123 (5.3%)	7 (17.1%)	10 (23.3%)	< 5

An ICSR may report more than one term; outcomes are reported by terms. ^†^“Recovering” states an undefined process of returning to a normal state at the time of reporting

Among the 29 reports on renal vascular and ischemic conditions, dosing of tranexamic acid was available for 17 reports (59%): in one ICSR 500 mg, in eight ICSRs 1 g, in five ICSRs 2 g, in two ICSRs 3 g, and in one ICSR 5 g were mentioned, respectively. Twenty-eight (97%) ICSRs reported patient outcome: One patient died, three did not recover, five did recover with a sequela, three were still recovering at the time of report, five recovered, and 11 had an unknown outcome.

### Statistical analysis

The results from the disproportionality analyses are presented in Table 2. Calculations showed a statistically significant signal for outcomes among users of tranexamic acid compared to any other drug in the database. A 32.6-fold increase in odds of reporting for renal vascular and ischemic conditions (ROR: 32.62, 95% CI: 22.50–47.29), a 2.5-fold increase for pulmonary thrombotic and embolic conditions (ROR: 2.52, 95% CI: 1.85–3.42), and a 2.7-fold increase for peripheral embolism and thrombosis (ROR: 2.52, 95% CI: 1.85–3.42) were observed.

**Table 2:** Reporting odds ratio (ROR) comparing individual case safety reports (ICSRs) on tranexamic acid to ICSRs on all other drugs within the WHO-UMC pharmacovigilance database. Database was filtered for female sex and ages 18 to 44 years

Adverse drug event	Tranexamic acid	Non-tranexamic acid*	ROR	95% confidence interval
Peripheral embolism and thrombosis	41	17,858	2.67	1.96–3.64
Pulmonary thrombotic and embolic conditions	42	19,408	2.52	1.85–3.42
Renal vascular and ischemic conditions	29	1035	32.62	22.50–47.29

*Applied filters were the same as for tranexamic acid (female sex, ages 18 to 44 years); entry dates were clipped to match the ICSRs for tranexamic acid (March 31, 1973)

*ROR* reporting odds ratio

## Discussion

In this analysis of spontaneous reports of adverse events, we identified that thrombo-embolic events accounted for approximately 5% of all reported outcomes for tranexamic acid among women of child-bearing age (18 to 44 years). Within 2245 included ICSRs on tranexamic acid, we identified 29 (1.3%) reports of adverse renal vascular and ischemic conditions, 42 (1.9%) reports of pulmonary thrombotic and embolic events, and 41 (1.8%) reports of peripheral embolism and thrombosis. Additionally, a signal of disproportionate reporting for renal vascular and ischemic conditions was observed with a reported median dose of 1 g of tranexamic acid.

We purposefully selected thrombo-embolic events as additional events of interest, alongside renal vascular and ischemic conditions, to put the calculated RORs into perspective. Because of its mechanism of action as a competitive inhibitor of fibrinolysis [21], tranexamic acid may be expected to increase the rates of thrombo-embolic events. In our disproportionality analysis, we observed increased odds of reporting for peripheral embolism and thrombosis and pulmonary thrombotic and embolic conditions of 2.7-fold (ROR: 2.67, 95% CI: 1.96–3.64) and 2.5-fold (ROR: 2.52, 95% CI: 1.85–3.42), respectively. A 32.6-fold (ROR: 32.62, 95% CI: 22.50–47.29) increase in odds of reporting for renal vascular and ischemic conditions, hence, marks an additional disproportionality.

Renal cortical necrosis is an ischemic destruction of the renal cortex caused by reduced arterial perfusion due to vascular spasm, microvascular injury, or intravascular coagulation [22, 23]. With its antifibrinolytic properties, tranexamic acid may harm the kidneys by favoring the formation of blood clots affecting kidney vessels and causing cortical necrosis. Hemorrhages and shock are characterized by hypovolemia and hypotension, which are risk factors for prerenal etiologies of kidney failure by themselves. The combination of fibrinolysis inhibition, hypovolemia, fibrinogen concentrates, and pregnancy-related coagulopathy [24] potentially has an adverse impact on the renal endothelium. Pregnant women and post-partum women are generally in a hypercoagulable state [25] and at an increased risk for thrombo-embolic events altogether [26, 27] due to elevated levels of factors VII, X, VIII, fibrinogen, and von Willebrand; an increase in prothrombin fragments and thrombin-antithrombin complexes; a reduction in protein S activity; and an acquired activated protein C resistance [28]. In our data, sixteen ICSRs (55%) additionally received sulprostone (Fig. 1), for which the product information labels adverse effects on renal function [29]. The concomitant use of the prostaglandin E2 analogue sulprostone may act as a mediating factor as it would impair the renal elimination of tranexamic acid [21] and increase tranexamic acid exposure.

The cases of renal corticosis as reported by Frimat et al. were observed with doses from 2 to 11 g in patients with post-partum hemorrhage (median 5 g) [10]. A recent RCT administered 1 g of tranexamic acid prophylactically to 1918 women, whereof 90% did not have post-partum hemorrhage [7]. There were no reported untoward thrombo-embolic events, including renal events or failure, potentially indicating dose-dependent effects and an interplay of disease and drug effects. Currently, doses of up to 2 g are recommended for the off-label treatment of post-partum hemorrhage [4, 21]. In our analysis, the median dose was 1 g, and 14 of the 17 ICSRs, that reported kidney damage and dosing of tranexamic acid, mentioned doses lower or equal to 2 g. We, therefore, feel that clinicians should be aware of renal complications even at low doses of tranexamic acid.

## Limitations

Analyses of pharmacovigilance data are prone to inherent limitations and need to be interpreted with care. Firstly, the calculated ORs are directly influenced by the number of reports sent to the database [14]. Health care professionals may be biased by current publications and discussions. Secondly, the quality of individual reports and clinical details provided may vary [30]. The reporters are primarily responsible for entering temporal relationships and initial assessments of causality; governance by national health authorities should, in theory, assure quality. Clinical details (e.g., lab values, comorbidities) are, if provided at all, restricted to the original reports and are not available through VigiBase. Hence, renal insufficiency was not available for our assessment, a predisposing factor for kidney failure and an important determinant for the renally excreted tranexamic acid exposure [31]. Thirdly, aggregated data on ICSRs does not include information on concomitant diagnoses, hindering assessments of potential confounding by diseases. Fourthly, the used ICSRs and numbers may have described incidents where the drugs were not used in obstetrics or for post-partum hemorrhage (e.g., menorrhagia, postoperative hemorrhage). Contrary to adverse reactions, indications are not amended by codes and are, hence, not possible to effectively process in VigiBase. We aimed to generate relevance for the obstetric population by restricting the search to female patients between 18 and 44 years of age. However, different indications may have been present in our final dataset. In culmination, our results should only be used as one singular piece of evidence and a safety signal to raise awareness. Disproportionality measures of spontaneous reports are not suited for causal inferences [32].

## Conclusion

We hereby report a disproportionality in reporting for tranexamic acid and renal vascular and ischemic conditions (e.g., renal cortical necrosis) in women aged 18 to 44 years. There is a need for observational studies investigating the causal relationship between tranexamic acid and rare adverse drug reactions. Until then, the benefits of tranexamic acid in acute situations such as post-partum hemorrhage should be weighed against its potential risks. This benefit-risk ratio may change with delays in treatment and may be different for prophylactic use.

## Data Availability

The data that support the findings of this study are available from WHO-UMC but restrictions apply to the availability of these data, which were used under special access for the current study, and so are not publicly available.
